# The hydrothermal processing of iron oxides from bacterial biofilm waste as new nanomaterials for broad applications†

**DOI:** 10.1039/c8ra07061j

**Published:** 2018-10-11

**Authors:** Le Yu, Diana N. H. Tran, Peter Forward, Martin F. Lambert, Dusan Losic

**Affiliations:** School of Chemical Engineering, The University of Adelaide Adelaide SA 5005 Australia dusan.losic@adelaide.edu.au; ARC Graphene Enabled Industry Transformation Hub, The University of Adelaide Adelaide SA 5005 Australia; SA Water South Australia 5005 Australia; School of Civil, Environmental and Mining Engineering, The University of Adelaide Adelaide SA 5005 Australia martin.lambert@adelaide.edu.au

## Abstract

Iron oxides and their hydroxides have been studied and analysed with properties of their mutual transformations under different hydrothermal conditions being indicated. Amorphous bacteria nanowires produced from biofilm waste were investigated under the influence of pH at a fixed duration (20 h) and reaction temperature (200 °C). The morphology, structure, and particle size of the transformation of hematite (α-Fe_2_O_3_) was obtained and characterised with SEM, XRD, FTIR, and particle sizer. The optimal conditions for the complete conversion of amorphous iron oxide nanowires to crystalline α-Fe_2_O_3_ is under acidic conditions where the pH is 1. The flower-like α-Fe_2_O_3_ structures have photocatalytic activity and adsorbent properties for heavy metal ions. This one-pot synthesis approach to produce α-Fe_2_O_3_ at a low cost would be greatly applicable to the recycling process of biofilm waste in order to benefit the environment.

## Abstract 2

Large production of crystalline, nanoflower hematite can be achieved under hydrothermal conditions from bacterial biofilm waste.

## Graphical abstract

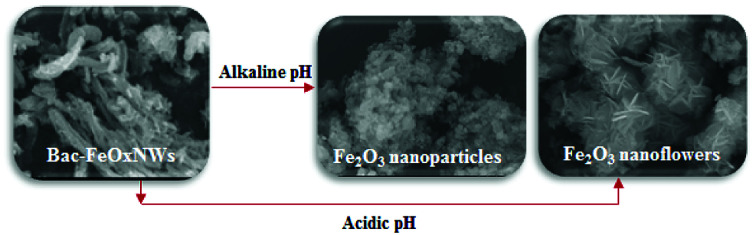

## Introduction

Nanomaterials have drawn a great deal of attention from both the public and scientific community due to their characteristic properties, which are dependent on their morphology, particle size and assembly patterns.^1^ Many works have tried to establish effective and feasible synthesis methods in order to control their assembly behaviours and morphology to further tailor their properties.^2^ One-dimensional (1D) nanostructures, such as nanowires, are interesting materials that can be naturally sourced from bacteria biofilms (Bac-FeOxs) which are abundant in South Australia (Australia). This versatile produced biofilm waste is found in the groundwater pumping system pipelines of the Murray River that require periodic cleaning to stop clogging from the accumulation of salt and bacteria (Mariprofundus ferrooxydans). This biofilm waste is comprised of ferromagnetic iron oxide wires with many unique properties. Our recent works have shown that they can be used as an efficient magnetic photocatalyst due to their structural performance under visible light irradiation, which could be applicable for cancer therapy^3,4^ and also be used as an adsorbent for the removal of arsenic in wastewater.^5^ Their wide usage in paints, coatings, cosmetics, magnetic and construction materials is also due to their chemical stability, low cost, high durability and high strength.^6^ A different study has shown that by catalysing hydrogen peroxide for the generation of free-radicals the biofilm can degrade, and also hinder its accumulation on human teeth.^7,8^ Some of the common methods to synthesize iron oxides are surfactant mediated-precipitation, chemical precipitation, electro-deposition and micro-wave assisted hydrothermal techniques. However, these methods are limited as the particle size and morphology cannot be tailored and applied to certain functions.^9^ Hydrothermal reduction is a simple method that can easily be implemented in industry as it requires only a ‘one-pot’ reaction. The transformation from an amorphous to a crystalline structure under thermal annealing has been demonstrated to give the nanowire a unique crystal-phase configuration without changing its morphology.^2^ Therefore, the recycling of this biofilm waste not only would decrease pollution and environmental waste but would also be a useful regenerative source for potential applications which is beneficial for the environment, economy and society.

Iron oxide (such as magnetite, Fe_3_O_4_) pigments have numerous desirable attributes as colouring agents, especially in cosmetics, as they can range in colours with high tinting strength. In addition, they are extremely stable (no fading and no bleeding) and are highly resistant. There are two major methods used to produce these iron oxides. One is to oxidize the iron metal with water and the other is to reduce hematite (Fe_2_O_3_) with hydrogen (H_2_) as shown in eqn (1) and (2), respectively:^10^

1 Fe + 4Fe_2_O_3_ = 3Fe_3_O_4_

2 3Fe_2_O_3_ + H_2_ = 2Fe_3_O_4_ + H_2_O

The reduction of Fe_2_O_3_ to Fe_3_O_4_ needs to be synthesized at both elevated pressure and temperature when it is in the presence of water. Both reactions need to be performed under hydrothermal conditions.

Iron oxides and their hydroxides have been studied and analysed with indicating properties of their mutual transformations under different hydrothermal conditions. Most of their transformations are set examples of a phenomenon called topotaxy.^11^ This phenomenon signifies the change of one solid crystalline phase to another form. It is believed that both two phases share a fixed structural relationship with one another. Within the iron oxide system, most transformations are well known, which have been examined and analysed in rational crystallochemical methods.^12^ The major six crystal structures of oxides and hydroxides iron are hematite, magnetite, maghemite, wustite, goethite and lepidocrocite. They are composed of various stackings of oxygen or hydroxyl sheets combined with different arrangement of iron ions in tetrahedral or octahedral spaces.^11^ Therefore, this structure could impact on the properties of one another within the iron oxide system. When the sheets are stacked differently, the structure will transform and automatically rearrange with a distinct stacking formation, where each sheet will share a strong link associated with each other. For the formation of a particular iron oxide, the annealing temperature and pH during the hydrothermal process is critical as this will affect the crystal structure of the material. Table 1 summarizes the different formation requirements of each oxide and hydroxide of iron under hydrothermal conditions. However, it is unclear where the optimised conditions are for the different iron oxide formation due to the very few and inconclusive available data in the current literature whether it is the time, temperature, or pH.

**Table 1:** The different properties of the five different oxides and hydroxide of iron under hydrothermal conditions^9,13,14^

Iron oxide	Colour	Crystal shape	Source	pH	Temperature	Time of reaction
Hematite	Red	Hexagonal prisms	Acidic: Fe(iii) solution with Fecl_3_ or Fe(NO_3_)_3_ solution base: amorphous iron(iii)hydroxides^12^	Acidic: 1–2 base: 8–10	Acidic >100 °C base: 100–200 °C	Acidic: several days base: -
Goethite	Brownish reddish yellow	Needle, laths	Acidic: Ferrihydrite precursor base: amorphous iron(iii)hydroxides^12^	Acidic: 1–2 base: 10.5–10.8^7^	Acidic: room temp base: 70 °C	Acidic: 50 days base: 60 h
Maghemite	Red to brown	Cubes	Heating synthetic lepidocrocite or synthetic magnetite	—	250 °C	2 h
Magnetic	Black	Cubes	Mixed solution of Fe(iii) and Fe(ii) with the ratio of 2	Base: 9–10	90 °C	30–60 min
Lepidocrocite	Reddish yellow	Laths	Slow hydrolysis of an acidic Fe(iii)	Ideal at 6.7–6.9 or <7.5–8	Room temp	2–3 h

To our knowledge there is no study on the hydrothermal reduction of iron oxides from Bac-FeOxs, which we present here. The aim of this work is to demonstrate for the first time the hydrothermal processing of iron oxides from biofilm waste in a ‘one-pot’ synthesis reaction. The conversion of hematite was targeted as it is the most important ore of iron. The temperature and pH of the hydrothermal process were optimised and the changes in structure were analysed. The synthesis method is simple and free of toxic wastes which can be scaled for industry.

## Experimental

### Materials and chemicals

Bacterial biofilms (Bac-FeOxs) were provided by SA Water (South Australia, Australia). Hydrochloric acid (HCl, 35%) and ammonium solution (NH_4_OH, 30%) were purchased from Chem-Supply (Australia). All chemicals were used directly without further processing. Distilled (DI) water was used throughout the study, unless otherwise stated.

### Purification and hydrothermal reduction of Bac-FeOxs

Bac-FeOxs were purified by washing the samples multiple times with DI water with a centrifuge (Sigma 3–18, John Morris, Australia) at 4200 rpm for 20 min until the conductivity (Thermo Scientific, Orion Star A212, Australia) of the samples reached that of DI water. This indicated that the salt impurities were successfully removed from the samples. X-ray diffraction (XRD, Rigaku MiniFlex 600, Japan) was also used to confirm the purity of the samples when no salt (*i.e.* sodium chloride, NaCl) peaks were measured. The samples were then dried at 60 °C in an oven overnight. For the hydrothermal process, 2.5 g of the purified Bac-FeOxs (P-Bac-FeOxs) were homogeneously mixed in 40 ml DI water for 30 min and the pH of the solution was adjusted from to 1–6 (HCl) or 8–10 (NH_4_OH) before transferring into a 50 ml Teflon-lined autoclave. The temperature of the reaction was fixed at 200 °C and was kept for 20 h. After the reaction the samples were washed with DI water (2×) for 10 min in a centrifuge (4200 rpm) then dried overnight at 60 °C in an oven and stored at 25 °C.

### Structural characterisation

The synthesised materials including the P-Bac-FeOxs were characterised by several analytical techniques such as, scanning electron microscopy (Hitachi SU1510, Japan) at an accelerating voltage of 30 kV, Fourier transform infrared (FTIR, Nicolet 6700 Thermo Fisher, Australia) spectroscopy scanned from 4000–400 cm^−1^ in transmission mode, and XRD measurements were collected in the range of 2*θ* = 20–80° (scan rate of 10 °C min^−1^). Particle size distribution (PSD) of all the materials was performed on a Malvern Mastersizer 2000 (Ata Scientific Pty Ltd, Australia).

## Results and discussion

### Characterisation of Bac-FeOxs

**Fig. 1:**
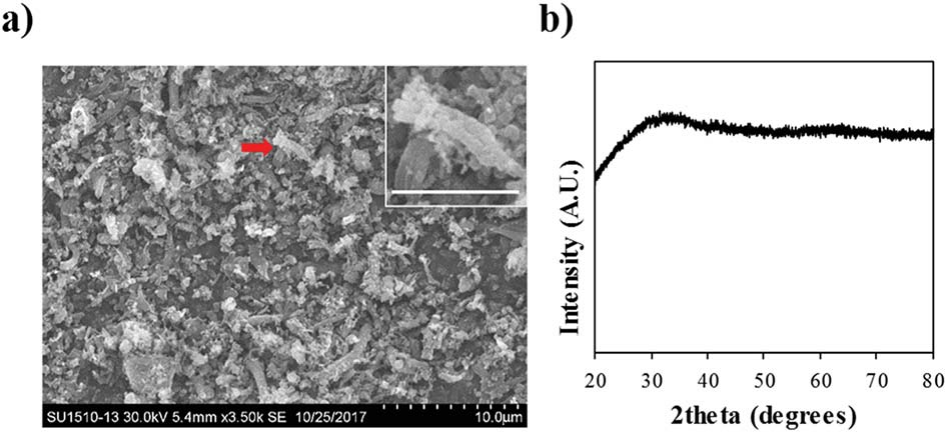
(a) Low resolution SEM image and (b) XRD spectra of purified Bac-FeOxNWs. Inset is a high-resolution SEM of the twisted nanowires indicated by the red arrow (scale bar = 3 μm).

An SEM image of P-Bac-FeOxs used in this work is presented in Fig. 1a. The image shows that the morphology of the P-Bac-FeOxs is comprised of broken and clustered nanowires <10 μm in size. The inset shows a clearer image of a bundled of nanowires. XRD confirmed the amorphous structure of the Bac-FeOx nanowires (Bac-FeOxNWs) (Fig. 1b) and removal of salt impurities from the washing process, which is in good agreement with the literature (Fig. S1†).

### Effect of pH and mechanism

**Fig. 2:**
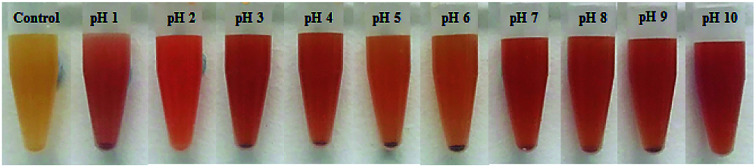
Photographs of Bac-FeOxNWs before reaction (control) and as a function of pH (1–10) after the hydrothermal process.

After the reaction, the colours of the synthesised Bac-FeOxNWs were observed as that was the first indication that a phase transformation had occurred. Fig. 2 distinctly shows the transition in colour from pH 1 to 10 compared to the control (purified Bac-FeOxNWs, yellow colour), which can be classed into 4 groups. Bac-FeOxNWs-pH 1 is slightly pink, Bac-FeOxNWs-pH 2 is orange, Bac-FeOxNWs-pH 3–4 and 7–10 is a deeper red colour, whereas Bac-FeOxNWs-pH 5–6 is light orange.

**Fig. 3:**
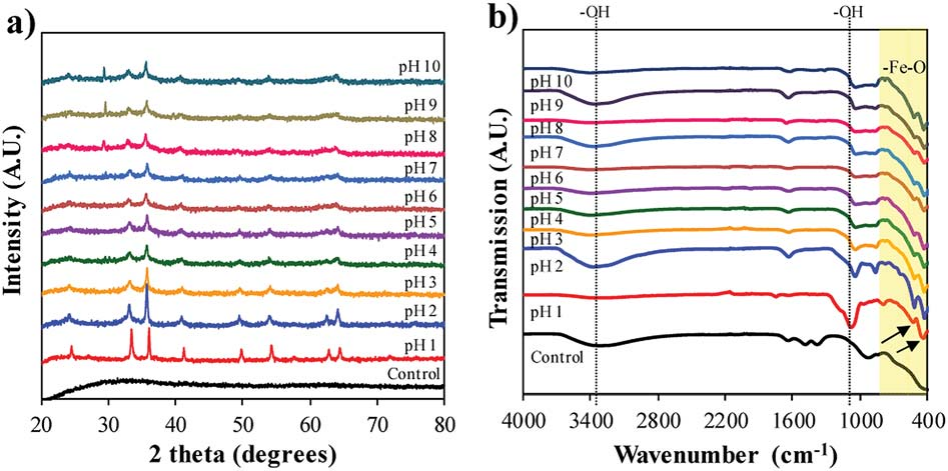
(a) XRD and (b) FTIR spectra for Bac-FeOxNWs before (control) after the hydrothermal process from pH 1 to 10.

Fig. 3a displays the XRD spectra of the different crystalline forms of the Bac-FeOxNWs from pH 1 to 10. Among these curves, the sharp and intensity peaks on pH 1 confirm the highly crystalline form of iron oxide.^23^ The peaks that appear at 2*θ* = 24.2, 33.2, 35.7, 42.5, 49.5, 54.1 and 64.2° corresponds to the characteristic peaks of α-Fe_2_O_3_ (JSPDS card no. 89-2810, Fig. S2a†).^15,16^ The sharp intensity peaks also suggests the high purity of the iron oxide production.^23^ The peaks on pH 2 and 3 are slightly less intense than pH 1, but still presents the typical peaks. On the other hand, the spectra of pH 4 and pH 5 weakly displays only four of the typical peaks at 2*θ* = 33.2, 35.7, 42.5 and 54.1°. The less distinct and weak peaks in pH 6 and 7 (2*θ* = 33.2, 35.7°) shows that the pH is not ideal for the complete formation of crystalline iron oxides. At pH 8–10 the XRD peaks are also less well-defined, although there is a phase transition occurring. The successful transformation of the amorphous Bac-FeOxNWs to α-Fe_2_O_3_ is under acidic conditions at 200 °C for 20 h is greater than in alkaline conditions at the same temperature and duration. The optimal hydrothermal condition of iron oxides production is pH 1 due to the higher crystalline form obtained.

FTIR spectra shown in Fig. 3b correlates with the findings interpreted from the XRD data. From the purified (control) and synthesised Bac-FeOxNWs curves (pH 1–10), the broad bands at 3400 cm^−1^ and narrow peaks at 1640 cm^−1^ correspond to the stretching –OH groups from the angular deformation of water.^17^ From pH 1–6, two typical bands at 470 cm^−1^ and 540 cm^−1^ are observed, which indicate the vibrations of metal and oxide of Fe–O, which is the form of the hematite phase (Fig. S2b†).^18–20,23^ However, these two peaks in Bac-FeOxNWs-pH 1, 2 and 3 are much sharper and narrower than those at pH 4–10, confirming the partial and weak transformation of the crystal iron phase. In addition, there is a medium and broad band approx. at 1080 cm^−1^ in both samples that may be attributed to atmospheric CO_2_, which commonly occurs in 20 h hydrothermal treated nano-particles.^16,24^ The hydrothermal process performed at pH 1–2 provide greater performance for synthesis of crystalline iron oxides.

**Fig. 4:**
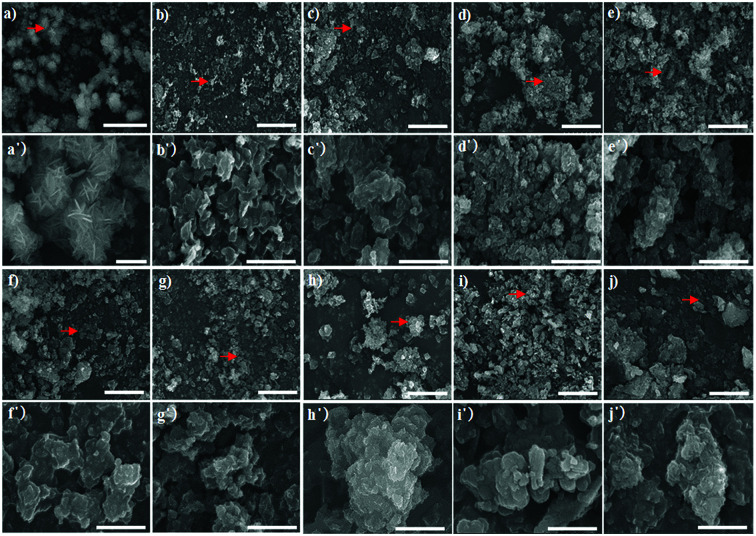
SEM images of Bac-FeOxNWs performed at pH 1–10 imaged at high (a–j, scale bar = 10 μm) and low (a′–j′, scale bar = 2 μm) resolutions, respectively. The red arrows shown are the areas imaged for the high-resolution SEM.

SEM images of the synthesised Bac-FeOxNWs taken at low and high magnifications further provide evidence for the difference in the nanostructure of each hydrothermal condition. Fig. 4(a–a′) shows the complete transition from the amorphous structure into high crystalline flower-like structure. These flower-like nanostructures have well-preserved hierarchical patterns of α-Fe_2_O_3_.^21,22^ At high SEM magnification, the exterior of each of the nanostructure is composed of irregular sheets. This flower-like nanostructure is attributed to the presence of HCl in the solution. During the reduction process, iron oxide and HCl react to form iron chloride (FeCl_3_). The FeCl_3_ solution is the key factor that contributes to the flower-like formation of α-Fe_2_O_3_ under a heating temperature of 200 °C 20–24 h.^21^Fig. 4(b–b′) shows clusters of wrinkled nano-sheets starting to form at pH 2, it is due to the fact that loose and irregular clusters occur under this hydrothermal condition. This structure is maintained at pH 3 (Fig. 4(c–c′)) with a similar show of irregular clusters where the breakdown of the structure begins, as observed at pH 4–5 (Fig. 4(d–d′) and (e–e′)). From pH 6 to 10, the structure begins to gather to form irregular and large microparticles, which is the transition from the early stage to the synthesis.^25^ Under highly alkaline conditions from pH 8–10 these irregular and large clusters become tightly-packed and well-shaped forms, especially in Fig. 4(h–h′).^26^Fig. 4(i and j) are the period of transition from the irregular forms into more crystalline structures again. Both pH 1 and pH 10 can provide well crystalline forms of iron oxide, however the acidic condition of pH 1 gives better performance with high crystallization of the flower-like forms.

**Fig. 5:**
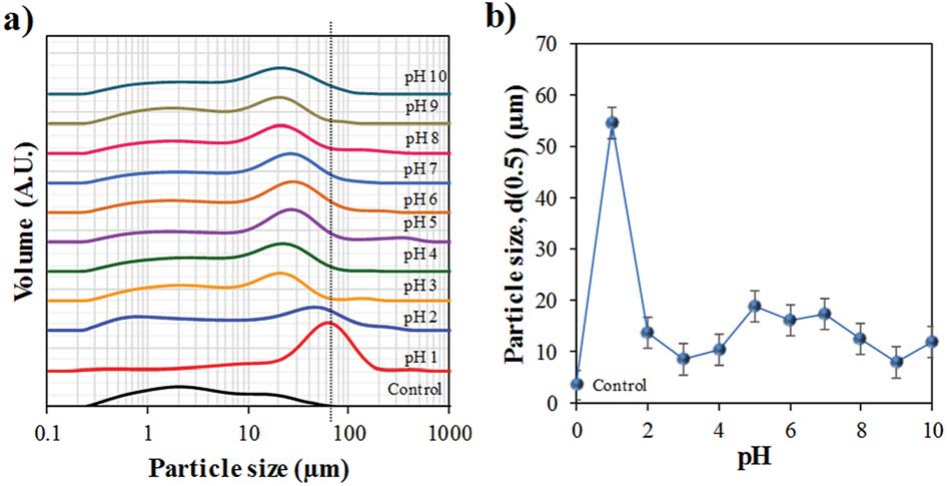
Particle size distribution (PSD) of the purified (control) and synthesised Bac-FeOxNWs from pH 1–10 after the hydrothermal process.

Particle size distribution of the raw and synthesised materials after the hydrothermal process are present in Fig. 5a. The Bac-FeOxNWs (control) displays a broad PSD ranging from 0.2 to 80.0 μm,^27^ which is expected due to the presence of many broken nanowires (Fig. 1a). As the nanowires are hydrothermally treated under high temperatures and in an acidic solution their morphology begins to change. The Bac-FeOxNWs synthesised at pH 1 displays a narrow PSD with larger particles (*d*(50) = 54.5 μm) compared to the broad PSD (twin peaks) at pH 2. With increasing pH from 3 to 4, the flower-like nanostructures break down into small particles from 13.8 to 10.4 μm, respectively. Around pH 5–7, the particle size of the synthesised Bac-FeOxNWs are consistent (*d*(50) = 17.2 μm) as the broken down particles start to form large clusters (Fig. 4(e–g)). Under the hydrothermal treatment in an alkaline condition, the clusters become well-packed and tightly bound from pH 8 to 10, hence a decrease in the particle size to 12.0 μm. When the powdered dispersions (Fig. 2) were re-examined after 24 h, it was noticed that all the particles at pH 1 had settled down compared to the well-dispersed stable solutions of all the other pHs. This result further confirms the formation of the larger particles at pH 1 (Fig. 5b) and is complemented by the SEM characterisation.

The study of Mohapatra and Anand^9^ shows that the only reaction product formed is hematite in the pH range of 0.8–2.6, however, this is contradictory to the results that claim that the iron product is formed greater under basic pH.^14^ The difference in results is related to the starting materials used to form α-Fe_2_O_3_. Majority of the studies use the iron chemical precursors to form the end product, whereas in this work we start with the natural amorphous iron oxide instead. Considering the XRD, FTIR, SEM and PSD results, the optimal hydrothermal treatment is under acidic conditions for Bac-FeOxNWs at a reaction temperature of 200 °C with pH 1 and a reaction time of 20 h for the complete transformation of α-Fe_2_O_3_. The reaction is successfully reduced due to the pink coloured powder that is obtained as illustrated in Fig. 2 than the majority of orange powders achieved for the other pH values. Although, red powders were obtained under alkaline pHs their crystalline structures are weak and are not fully developed.

This ‘nucleation, growth and ostwald ripening’ behaviour is common for the synthesis of iron oxides under different conditions (*e.g.* time, temperature, environment).^28–32^ Under hydrothermal conditions, starting at neutral pH 7, by decreasing pH (acidic) the system shows that the particles grew through Ostwald ripening, while at basic pH the particles underwent continuous nucleation and growth to form aggregates,^23,25^ which confirms the SEM and PSD data.

### Applications

**Fig. 6:**
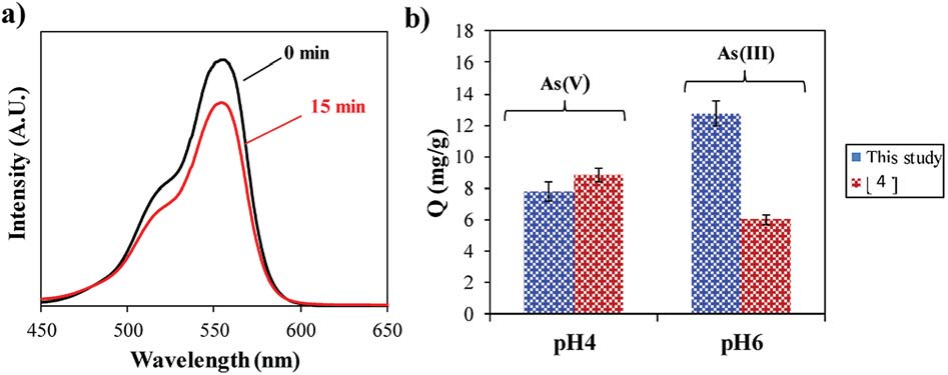
(a) UV-Vis spectra of the degradation of RhB at 15 min, and (b) and adsorption capacity (*Q*) of As(iii) and As(v) ions after 90 min.

The potential use of α-Fe_2_O_3_ produced at the optimal acidic condition (pH 1) from the hydrothermal reduction process was tested with 2 different applications. Photodegradation of Rhodamine B (RhB, Sigma Aldrich, Australia) was performed on the synthesized product. Details of the experimental set up is described elsewhere.^4^ The data shows that after 15 min of exposure to visible light the amount of RhB in the solution had decreased as measured with UV-vis spectroscopy (Shimadzu UV-Vis 1601, Japan) (Fig. 6a). This suggests that both the aromatic rings and chromophores of RhB have been destroyed, therefore demonstrating that the synthesized α-Fe_2_O_3_ material has photocatalytic properties. The product was also used as an adsorbent for the removal of heavy metal ions (*e.g.* arsenic) as illustrated in Fig. 6b. The adsorption study was carried out at room temperature (22 °C) with an initial concentration of 10 mg ml^−1^ using 10 mg of adsorbent. The experimental procedure is given elsewhere.^5^ Comparative studies with our previous work^4^ shows similar adsorption capacity (*Q*) for As(v) (∼9 mg g^−1^) but a double improvement in the adsorption of As(iii) (13 mg g^−1^) performed at pH 4 and 6, respectively, after 90 min. This could be related to the greater surface area of the crystalline nano-flowers compared to the untreated Bac-FeOxNWs, which was smaller in particle size.

## Conclusions

In this work, we demonstrated for the first time the hydrothermal reduction of iron oxides from bacteria biofilm waste under acidic and basic conditions at a fixed reaction temperature and time. Comparing the results that were achieved at basic pH, the evidence strongly shows that the optimal condition for the transformation of amorphous iron oxide nanowires to crystalline α-Fe_2_O_3_ is under acidic condition where the pH is 1. Highly crystalline nano-flower structures were obtained with a large particle size of 54.5 μm. We also showed that the nano-flower α-Fe_2_O_3_ structures have photocatalytic activity and adsorbent properties for heavy metal ions. This one-pot synthesis reaction makes this a simple waste-free method which requires low cost to produce, thereby providing excellent opportunity to recycle this abundant waste material for environmental and catalysis applications.

## Conflicts of interest

There are no conflicts to declare.

## Supplementary Material

RA-008-C8RA07061J-s001
