# Supplementary material for: The hydrothermal processing of iron oxides from bacterial biofilm waste as new nanomaterials for broad applications
Source: RSC Adv. 2018 Oct 11;8(61):34848–52. doi: 10.1039/c8ra07061j (PMC9087643; doi:10.1039/c8ra07061j)
Supplement: RA-008-C8RA07061J-s001 [file RA-008-C8RA07061J-s001.pdf]

Electronic Supplementary Material (ESI) for RSC Advances.
This journal is © The Royal Society of Chemistry 2018

Supplementary Information

The hydrothermal processing of iron oxides from bacterial biofilm waste as new nanomaterials for broad applications

Le Yu[a], Diana N.H. Tran[ab], Peter Forward[c], Martin F. Lambert[d], and Dusan Losic*[ab]

[a] *School of Chemical Engineering, The University of Adelaide, Adelaide, SA 5005, Australia. E-mail: dusan.losic@adelaide.edu.au.*
[b] *ARC Graphene Enabled Industry Transformation Hub, The University of Adelaide, Adelaide, SA 5005, Australia.*
[c] *SA Water, South Australia, 5005, Australia.*
[d] *School of Civil, Environmental and Mining Engineering, The University of Adelaide, Adelaide, SA 5005, Australia. Email: martin.lambert@adelaide.edu.au.*

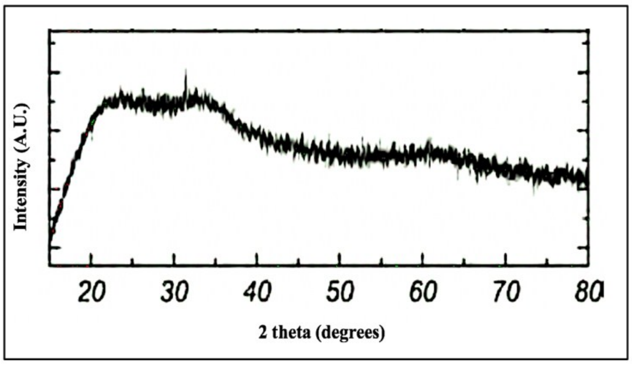

Figure S1. XRD spectra of amorphous iron oxide[1]

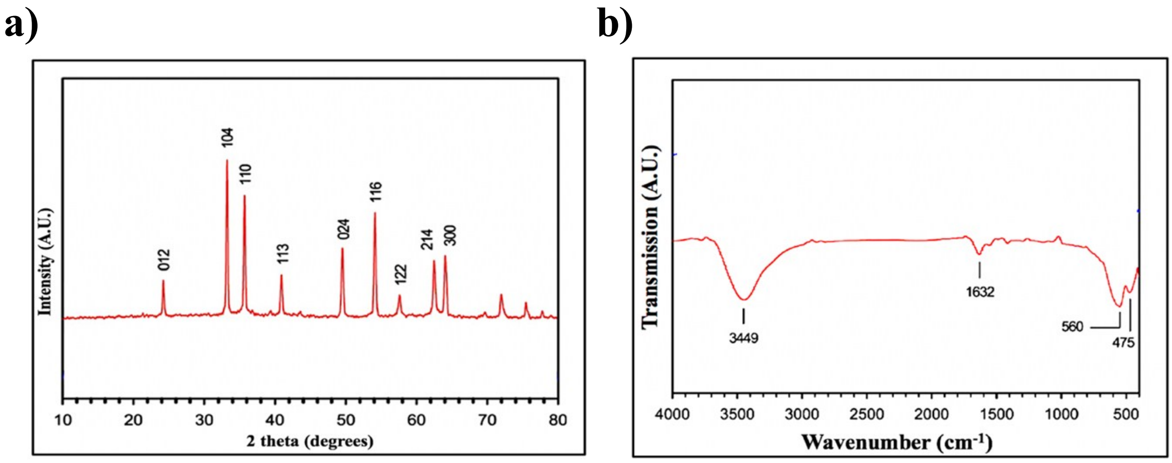

Figure S2. (a) XRD and (b) FTIR spectra of crystalline iron oxide[2,3]

References

1. L. Wang, T. Kumeria, A. Santos, P. Forward, M. F. Lambert and D. Losic, *ACS Appl. Mater. Interfaces*, 2016, **8**, 20110-20119.
2. M. Sinha, S. Sahu, P. Meshram, L. Prasad, and B. Pandey, *Powder Technology*, 2015, **276**, 217.
3. K. Supattarasakda, K. Petcharoen, T. Permpool, A. Sirivat and W. Lerdwijitjarud, *Powder Technology*, 2013, **249**, 353-359.
